# Construction of a virtual *Mycobacterium tuberculosis* consensus genome and its application to data from a next generation sequencer

**DOI:** 10.1186/s12864-015-1368-9

**Published:** 2015-03-20

**Authors:** Kayo Okumura, Masako Kato, Teruo Kirikae, Mitsunori Kayano, Tohru Miyoshi-Akiyama

**Affiliations:** Department of Animal and Food Hygiene, Obihiro University of Agriculture and Veterinary Medicine, Inada-cho, Obihiro, Hokkaido 080-8555 Japan; Department of Infectious Diseases, National Center for Global Health and Medicine, 1-21-1, Shinjuku-ku, Tokyo, 162-8655 Japan

**Keywords:** *Mycobacterium tuberculosis*, Consensus sequence, Virtual typing, Phylogenetic analysis, SNP concatemer

## Abstract

**Background:**

Although *Mycobacterium tuberculosis* isolates are consisted of several different lineages and the epidemiology analyses are usually assessed relative to a particular reference genome, *M. tuberculosis* H37Rv, which might introduce some biased results. Those analyses are essentially based genome sequence information of *M. tuberculosis* and could be performed *in sillico* in theory, with whole genome sequence (WGS) data available in the databases and obtained by next generation sequencers (NGSs). As an approach to establish higher resolution methods for such analyses, whole genome sequences of the *M. tuberculosis* complexes (MTBCs) strains available on databases were aligned to construct virtual reference genome sequences called the consensus sequence (CS), and evaluated its feasibility in *in sillico* epidemiological analyses.

**Results:**

The consensus sequence (CS) was successfully constructed and utilized to perform phylogenetic analysis, evaluation of read mapping efficacy, which is crucial for detecting single nucleotide polymorphisms (SNPs), and various MTBC typing methods virtually including spoligotyping, VNTR, Long sequence polymorphism and Beijing typing. SNPs detected based on CS, in comparison with H37Rv, were utilized in concatemer-based phylogenetic analysis to determine their reliability relative to a phylogenetic tree based on whole genome alignment as the gold standard. Statistical comparison of phylogenic trees based on CS with that of H37Rv indicated the former showed always better results that that of later. SNP detection and concatenation with CS was advantageous because the frequency of crucial SNPs distinguishing among strain lineages was higher than those of H37Rv. The number of SNPs detected was lower with the consensus than with the H37Rv sequence, resulting in a significant reduction in computational time. Performance of each virtual typing was satisfactory and accorded with those published when those are available.

**Conclusions:**

These results indicated that virtual CS constructed from genome sequence data is an ideal approach as a reference for MTBC studies.

**Electronic supplementary material:**

The online version of this article (doi:10.1186/s12864-015-1368-9) contains supplementary material, which is available to authorized users.

## Background

Tuberculosis is one of the most prevalent and deadly bacterial infections affecting humans, with almost 9 million new cases worldwide and more than 1.4 million deaths in 2010 [1]. It has been estimated that 310000 patients newly diagnosed with pulmonary tuberculosis in 2011 were infected with multidrug-resistant (MDR) bacteria [2], with 9% of these patients, living in 84 countries, having extensively drug-resistant (XDR) tuberculosis [3]. Moreover, the World Health Organization (WHO) has estimated that 350000 of the 1.4 million deaths per year from tuberculosis are associated with HIV coinfection [4].

A variety of molecular typing methods have been used to classify *M. tuberculosis* strains for epidemiological studies, including assessment of the presence of the IS6110 restriction fragment length polymorphism (RFLP) [5], spoligotyping, analysis of mycobacterial interspersed repetitive unit-variable number tandem repeats (MIRU-VNTR) [6] and large sequence polymorphisms (LSPs) [7,8]. Their target sequences are mobile elements (e.g. IS6110), repetitive sequences (e.g. spoligotyping and MIRU-VNTR) and relatively long sequence polymorphisms (at least 7 bp), with many strains belonging to unrelated lineages possessing these DNA elements in common. According to the SpolDB4 of the international online spoligotype database (http://www.pasteur-guadeloupe.fr/tb/bd_myco.html), clinical strains of *M. tuberculosis* obtained worldwide can be classified into 10 major groups [9]. Although it is useful to identify clonal lineage on the global scale, the discriminatory power of this method may not be sufficient for evaluation of genetically closely related isolates, including those from areas of tuberculosis outbreak. According to the SpolDB4, all *M. tuberculosis* strains belonging to the Beijing family, predominantly from Far-East Asia [10], share the same spoligotype patterns [9]. Over 70% of the clinical strains isolated in Japan were found to belong to the Beijing family [11]. Similarly, MIRU-VNTR genotyping [6,12], based on the typing of 12 MIRU loci, has become a global standard in the epidemiological typing of *M. tuberculosis*. Since its first use, many investigators have tried to find an ideal combination to further distinguish among genetically closely related strains [13,14]. This has led to the formulation of optimized sets, including a 15-locus system as the new standard for routine epidemiological discrimination and a 24-locus system as a high-resolution tool for phylogenetic studies [14]. Although these 15- and 24-locus VNTR locus systems have been utilized for first-line typing, they are insufficient in distinguishing among closely related strains of the Beijing family to define deep phylogenetic structures [15]. LSPs have been utilized to determine whether lineages of *M. tuberculosis* were associated with specific human populations [7,8]. Utilizing LSPs, MTBCs could be divided into at least 6 phylogeographical lineages, each associated with specific, sympatric human populations. Taken together, that conventional molecular typing methods for MTBC are limited in distinguishing among strain subtypes.

This limitation may be overcome by next generation whole genome sequencing (WGS) for genome-based epidemiology [16]. WGS is becoming the ultimate tool for diagnosing and typing pathogens, and has amplified the impact of molecular diagnostics on clinical microbiology [17]. The potential of WGS-based MTBC genotyping has started to be explored [18]. In phylogenetic analysis based on WGS data, sequence reads are mapped to a reference genome, usually H37Rv, and single nucleotide polymorphisms (SNPs) are detected and concatenated to generate an artificial genome sequence representing each isolate. This approach has been shown to be robustness and of high resolution [19-21].

In comparative genome or phylogenetic analysis, generated genomes or WGS data are compared to results from reference genomes. The *M. tuberculosis* strain H37Rv was the first sequenced in its entirety [22] and has been utilized extensively as the reference genome in these investigations. In these comparisons, the sequences of one or several newly sequenced genomes are compared to the sequence of a reference genome. If the reference genome used in these analyses does not contain a gene or marker possessed by the newly sequenced genome, this method cannot be used to determine the evolutionary fate of the genetic context of the latter.

The evolution of MTBC genomes has several unique features. Although several mycobacterium phages have been reported [23,24], they were found to evolve through mutation without acquiring any external genetic traits. This feature differs strikingly from that observed in conventional drug resistance bacteria such as *Pseudomonas aeruginosa* and *Escherichia coli* [25]. The nature of its evolution makes the MTBC genome highly stable, and the genetic diversity of MTBCs obtained in clinical settings is essentially restricted in SNPs and indels. Generally, mobile elements such as transposons, phages and plasmids are omitted from phylogenetic analysis because their rate of evolution differs from that of the intrinsic chromosome. MTBCs, however, are composed of several lineages, and the development of an artificial reference sequence of the entire MTBC genome, containing sequence information from all lineages, would be extremely useful. Although some SNPs and/or indels may be omitted or ignored when using a particular genome sequence of a real isolate as a reference, these SNPs and/or indels would not be missed in comparative genomics, especially in phylogenetic research.

In this study, we constructed a consensus whole MTBC genome sequence from the sequence data of 19 MTBC strains isolated and characterized through April 2012. For proof of concept, we compared the consensus and H37Rv genomes as reference standards for phylogenetic analysis, SNP detection and secondary analysis of WGS data. In addition, we found that spoligotype, VNTR type, LSP classification, Beijing type and antibiotic susceptibility profiles could be determined simultaneously, resulting in a virtual diagnosis in the absence of actual experiments except for WGS.

## Results

### Construction and features of a virtual *M. tuberculosis* consensus genome

We constructed two types of *M. tuberculosis* consensus genome sequences, one consisting of 13 *M. tuberculosis* strains and the *M. bovis* BCG, *M. africanum* and *M. canettii* strains shown in Table 1, and the other consisting only of the 13 *M. tuberculosis* strains*.* In this study, the former was used for further analysis, unless otherwise specified. Public databases such as DNA Data Bank of Japan do not accept virtual sequence for the registration. Thus, CSs constructed in this study are available as the supplemental data (Additional files 1, 2 and 3).

**Table 1:** ***Mycobacterium*** **strains used in this study**

**Species**	**Strain name**	**GenBank**	**Country**	**Genome inversion points**	**Comments**
*M.tuberculosis*	CCDC5079	CP001642.1	China		Drug-susceptible isolate belonging to the Beijing family.
	CCDC5180	CP001642.1	China		Multidrug-resistant clinical isolate.
	CDC1551	AE000516.2	USA		
	CTRI-2	CP002992.1	Russia		
	Erdman	AP012340.1	USA		isolated from sputum samples of patients
	F11	CP000717.1	South Africa		Predominant strain in South African epidemic
	H37Ra	CP000611.1.	China		An avirulent strain derived from its virulent parent strain H37
	H37Rv	AL123456.2	USA		
	KZN605	CP001976.1	South Africa	1. between 932051 and 932052. 2. between 3479594 and 3459595	Extensively drug-resistant clinical isolate
	KZN1435	CP001658.1	South Africa	1. between 931985 and 931986. 2. between 3479865 and 3479866	Multidrug-resistant clinical isolate
	KZN4207	CP001662.1	South Africa	1. between 932007 and 932008. 2. between 3476553 and 3476554	Drug-susceptible clinical isolate
	RGTB327	CP003233.1	India		isolated from sputum samples of patients
	RGTB423	CP003234.1	India		isolated from sputum samples of patients
*M. bovis* BCG	Mexico	CP002095.1	Mexico		
	Moreau RDJ	AM412059.2	Brazil		Brazilian vaccine strain
	Pasteur	AM408590.1	France		
	Tokyo 172	AP010918.1	Japan		
*M. africanum*	GMO41182	FR878060.1	Gambia		
*M. canettii*	CIPT 140010059	HE572590.1	France		

Although the concept of a consensus genome has long been recognized [26-30], no consensus genome had been determined for *M. tuberculosis*, largely due to difficulties in aligning these relatively large sized genomes. Despite its relatively stable and conserved genome, some *M. tuberculosis* strains have inversions and rearranged regions in comparison with H37Rv, making alignment more difficult. To overcome the inversions and rearrangements in *M. tuberculosis* strains, we performed genome rearrangement analyses using publicly available software Mauve [31], finding that the genomes of *M. tuberculosis* KZN605, KZN1435 and KZN4207 have large inversions, of 0.93–3.46Mbp (Table 1). IS6110 insertion sequences are located immediately adjacent to the flanking regions of all the inversion points, suggesting that the genome rearrangements observed in these 3 strains were driven by the insertion sequence. Manual correction of these inversions generated artificial KZN605, KZN1435 and KZN4206 genome sequences (KZN605_m, KZN1435_m and KZN4206_m, respectively), with the latter used to align the MTBC genomes. These procedures and employing a very fast and publicly available multiple sequence alignment software, MAFFT [32] allowed the successful alignment of the genomes of 19 MTBC strains and 13 *M. tuberculosis* strains. Merging the alignment results were carried out using sequence editing commercial software, Genetyx, although any software, which can handle multiple alignment data, should be suitable for the purpose. Two types of CSs were prepared with handling SNPs as majority rule or ambiguity rule. In this study, ambiguity sequence was used for further analysis. Insertion sequences derived from strain specific regions were all included in the CSs to increases the amount of sequence information.

The MTBC CS consists of one chromosome of 4991559 bp, with an average GC content of 64.8%. This genome was approximately 0.6 Mbp longer, because all sequence data from all strains used were merged into one sequence, and had a slightly lower GC content than the elements of the 19 strains used to construct the consensus genome. This artificially merged CS was intended for use as a reference genome in analyses of MTBC. Thus, instead of CDS extraction followed by annotation, homology analysis based on the corresponding gene sequences of *M. tuberculosis* H37Rv, which is extensively used as a reference genome, was performed to annotate the corresponding region in CS. Each region was assigned based on its CDS locus_tag (the Rv numbers), repeat regions, and rRNA and tRNA of H37Rv. All locus_tags of H37Rv were reflected in CS. This annotation resulted in features based on 4395 CDS (annotated as misc_features in CS) and repeat regions according with those H37Rv. Public databases do not accept virtual sequences. Thus the completely annotated sequence in addition to the alignments is available as additional data (Additional file 3).

Genome wide comparisons of *M. tuberculosis* strains have been performed extensively and repeatedly [33-35]. SNP concatenation is of the state of art methodology extensively used to analyze the phylogenetic relationship of bacterial genome [20,36]. We analyzed SNPs and indels in the CS reference genome in comparison with H37Rv to update fundamental information about polymorphism found in MTBC (Additional file 4: Tables S1 and Additional file 5 Table S2). The number of SNPs was higher in *M. canettii* than in the other mycobacteria (26068 vs <4000) (Table 2), suggesting a potential erroneous analysis when compared with a particular MTBC strain, such as H37Rv. For the analysis of indel, we chosen >5 bp length as the cut off of indels because indels shorter than 5 bp are overlapped many strains and detection of some them are highly depend on the alignment parameters. As reported previously, 152 of the 305 indels were located in the genes encoding the PE-PGRS and PPE family proteins, while 74 were located in intergenic regions (Additional file 5: Table S2). The position, length, annotation and MTBC strains of all SNPs and indels are shown for further applications, such as searches for lineage specific markers.

**Table 2:** **Efficacy of SNP calling using the consensus sequence or H37Rv as the reference genome by comparing the number of SNPs detected**

**i) individual strains**			
**Species**	**Strain**	**vs H37Rv**	**vs consensus sequence**
*M. tuberculosis*	CCDC5079	632	198*
	CCDC5180	378	70*
	CDC1551	543	41*
	CTRI-2	140	15*
	Erdman	570	104*
	F11	343	129*
	H37Ra	44	38*
	H37Rv		12
	KZN605	16	9
	KZN1435	12	2
	KZN4207	26	0
	RGTB327	1242	127*
	RGTB423	2410	145*
*M. bovis* BCG	Mexico	13	0
	Moreau RDJ	136	22*
	Pasteur	20	0
	Tokyo 172	56	76*
*M. africanum*	GMO41182	876	152*
*M. canettii*	CIPT 140010059	24425*	560
Total SNP found in only one strain		31882	1700
	ii) group comparison		
	four BCG strains	1040	52*
	three KZN strains	121	15
	All SNP at least found in one strain	37589	3429

Based on SNP calls using MUMmer [58], the number of SNPs called uniquely in individual strains, and in groups of BCG and KZN strains, was determined. *The number of SNPs detected using the H37Rv and consensus sequences as reference were compared for each strain (i) or group of strains (ii) using Fisher's exact test, with significant differences indicated with asterisks (p < 0.0001).

### Comparison of the performance of the consensus sequence (CS) and H37Rv as the reference genome in the phylogenetic analyses

**Figure 1:**
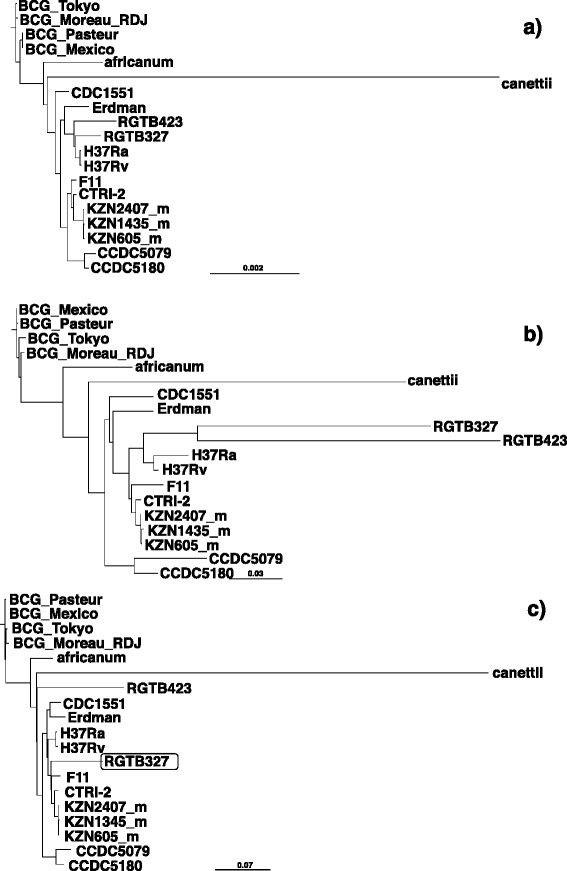
**Maximum-likelihood phylogenies based on whole genome and SNP concatenated sequence alignment.** Phylogenetic trees based on whole genome sequences **(a)**, SNP concatemers using CS as reference **(b)** and SNP concatemers using the H37Rv genome sequence as reference **(c)** were constructed using PhyML 3.0 [39]. Most probable trees were selected based on aLTR statics implemented in PhyML [39]. Isolates, clustered into different positions compared with the phylogenetic tree based on the whole genome sequences of *M. tuberculosis* strains RGTB327 and RGTB423, are indicated in the squares. For the KZN series, inversion-corrected sequences were used for the alignment and marked “_m”. aLTR statics values for each branch are shown. In Figure 1a, clusters of lineage 4 and 2 are indicated in the squares.

**Figure 2:**
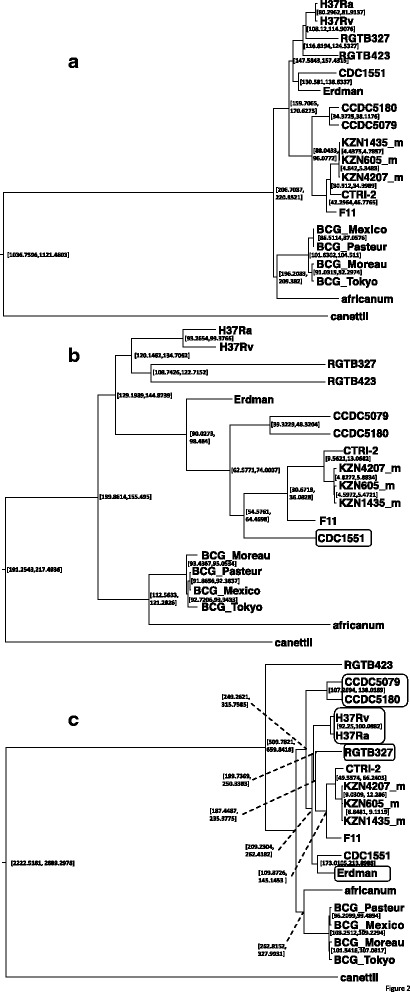
**Bayesian post-probable phylogenies based on whole genome and SNP concatenated sequence alignment.** Description of data: Phylogenetic trees based on whole genome sequence **(a)**, SNP concatemers using the consensus genome sequence as reference **(b)** and SNP concatemers using H37Rv genome as reference were constructed using BEAST [38]. All relevant parameters reached an effective sample size of >100, indicating good convergence of the chains. For each branch, 95% highest posterior density is shown with good support. Isolates, clustered into different positions compared with the phylogenetic tree based on the whole genome sequences of *M. tuberculosis* are indicated in the squares.

To show that CS was superior to the sequence of a particular strain in phylogenetic analysis in preparation of SNP concatemers, we first constructed phylogenetic trees based on concatenated SNP sites from the virtual consensus and H37Rv genome sequences. Two tree construction methods based on a maximum-likelihood (PhyML, [37]) and Bayesian MCMC (BEAST, [38] were used for the validation each other. Most probable trees were selected based on three methods; approximate likelihood-ratio test (aLRT) statistics [39] implemented in PhyML and combination of 9 statistical analysis implemented in CONSEL [40] in the maximum-likelihood methods, and 95% highest posterior density (HPD) in Bayesian MCMC. Tree topology was tested based on statistical methods implemented in CONSEL, and tree distance was quantified using Robinson–Foulds metric [41] implemented in treedist in the PHYLIP package. When compared with the phylogenetic tree based on whole genome alignment of MTBC (Figure 1a) as the gold standard, the tree based on SNP concatemers from CS (Figure 1b) showed essentially the same topology as the maximum-likelihood phylogenetic tree chosen by aLTR statics, whereas the tree based on the H37Rv sequence showed different positioning of RGTB327 and RGTB423 (Figure 1c). In Bayesian MCMC phylogeny chosen by 95% highest posterior density (HPD), compared with the phylogenetic tree based on whole genome alignment of MTBC (Figure 2a), the tree based on SNP concatemers from CS showed different positioning of CDC1551, while that from H37Rv showed different positioning of RGTB327, Erdman, CCDC5079, CCDC5180, H37Rv and H37Ra (Figure 2c). The same tendency was observed in phylogenetic trees chosen by combination of 9 statistical analyses in maximum-likelihood with bootstrapping (Additional file 6: Figure S1 and Additional file 7: Table S3). In all three trees chosen by different statistical methods, distance between tree based on whole genome alignment and SNP concatemers derived from CS was always smaller than that based on whole genome alignment and SNP concatemers derived from H37Rv (Table 3). These results indicated that SNP concatemers based on different reference sequences behave differently in phylogenetic analysis, emphasizing the critical importance of selecting the proper reference sequence, and CS is superior to H37Rv when it is used as the reference sequence in phylogenetic analysis of MTBC. We also compared the computational times required by these analyses (Table 4). Use of CS as the reference markedly reduced the times required for both the maximum-likelihood (5 vs 35 hr with bootstrapping) and Bayes MCMC (2 vs 22 hr) methods without a critical deterioration in tree topology when compared with whole genome alignment (Figures 1 and 2). The time difference observed could be explained essentially by two parameters, the size and quality of alignments. Alignment length based on whole genome, SNPs derived from CS and SNPs derived from H37Rv is 5.0 Mbp, 21,425 bp, and 52,203 bp, respectively, and H37Rv based SNP concatemers contain biased SNPs (see below). Reduced size and better quality of alignment seems to contribute the reduction of computational time.

**Table 3:** **Distance analysis among phylogenic trees constructed based on maximum-likelihood and Bayesian MCMC methods**

**Phylogenetic analysis**	**vs CS**		**vs H37Rv**	
	**Unrooted**	**Rooted**	**Unrooted**	**Rooted**
PhyML(aLRT)	2.88E-01	2.88E-01	5.95E-01	5.95E-01
PhyML(consel selected)	2.89E-01	2.89E-01	5.96E-01	5.96E-01
BEAST	1.68E + 03	1.20E + 03	2.61E + 03	1.91E + 03

To quantify the branch score distance between trees, Robinson and Fould test [41] implemented in treedist in the Phylip package was utilized. Both of unrooted and rooted scores were calculated.

**Table 4:** **Computational time for each phylogenetic analysis**

**Sequence type**	**PhyML3 (bootstrapping)**	**PhyML3 (aLRT)**	**BEAST ver.1.7**
Whole genome	34h59min55sec	2min49sec	21h40min45sec
Consensus sequence	4h50min9sec	1min24sec	1h47min24sec
H37Rv	19h24min38sec	2min37sec	2h38min31sec

For PhyML, two tree selection methods, 100 bootstrappings and aLRT were performed. In the bootstrap analyses, multithreading with 16 CPUs were utilized to reduce the computational time. For BEAST, 10 million samplings were performed for each analysis. Computational times were based on the log file of each analysis.

To obtain further insight on the behavior of SNP concatemers relative to on the different reference sequences, we analyzed the number of SNP called in individual strains using as reference the consensus or the H37Rv genome sequence (Table 5). We observed marked bias in the number of SNPs called in each strain when H37Rv was the reference. The number of SNPs was much higher in *M. canettii* than in the other strains. Large numbers of SNPs were also present in *M. tuberculosis* strains RGTB423, RGTB327, CCDC5079, CCDC5180, CDC1551 and Erdman, which behave differently in phylogenetic analyses based on their SNP concatemers (Additional file 7: Figure S1). Differences among strains in the number of SNPs were reduced when CS was used as the reference. Statistical analysis indicated that the rate of detection of SNPs unique to a particular strain was significantly higher using the consensus than using the H37Rv sequence as a reference (Table 2). The only exception was *M. canettii*, which showed a higher detection rate when compared with H37Rv. The BCG and KZN series, each consists of closely related strains, with individual strains having small numbers of unique SNPs. In comparing the number of detection of SNPs commonly found in BCG or KZN strains (Table 2), we found that detection of SNPs in BCG strains was greater using the consensus than the H37Rv sequence, although no significant difference was observed in detecting SNPs in KZN strains. These results indicated that SNP calling with CS makes possible the better detection of truly unique and crucial SNPs, which discriminate accurately among the strains.

**Table 5:** **Comparison of Illumina read mapping efficacy using clinical isolates derived from different lineages using Bowtie2 and SAMtools**

**i) Comparison of the numbers of mapped and unmapped reads to the H37Rv sequence or consensus sequence**								
	**LineAge**		**H37Rv**		**Consensus sequence**		**Subtraction of ratio (%)**	
		Mapping stringency*	**Local**	**End to end**	**Local**	**End to end**	**Local**	**End to end**
							**(CS minus H37Rv)**	
F092	EAI	mapped	681561	664376	684952	667250		
		unmapped	22261	39446	18870	36572		
		ratio (%)	96.837	94.395	97.319	94.804	0.482	0.408
J156	EAI	mapped	1680156	1650866	1689673	1658917		
		unmapped	40162	69452	30645	61401		
		ratio (%)	97.665	95.963	98.219	96.431	0.553	0.468
F038	Haarlem, LAM, X etc.	mapped	1024873	997301	1029625	1000714		
		unmapped	75113	102685	70361	99272		
		ratio (%)	93.171	90.665	93.603	90.975	0.432	0.310
F070	Haarlem, LAM, X etc.	mapped	858126	840921	861393	843463		
		unmapped	27822	45027	24555	42485		
		ratio (%)	96.860	94.918	97.228	95.205	0.369	0.287
J073	Haarlem, LAM, X etc.	mapped	1534315	1503494	1537891	1504256		
		unmapped	11979	42800	8403	42038		
		ratio (%)	99.225	97.232	99.457	97.281	0.231	0.049
J147	Haarlem, LAM, X etc.	mapped	847807	836269	849747	836489		
		unmapped	11775	23313	9835	23093		
		ratio (%)	98.630	97.288	98.856	97.313	0.226	0.026
F081	other non-Beijing	mapped	1004912	974425	1008107	976556		
		unmapped	43978	74465	40783	72334		
		ratio (%)	95.807	92.901	96.112	93.104	0.305	0.203
J020	other non-Beijing	mapped	1081365	1062537	1085065	1065704		
		unmapped	11293	30121	7593	26954		
		ratio (%)	98.966	97.243	99.305	97.533	0.339	0.290
J027	other non-Beijing	mapped	751633	741219	754861	744254		
		unmapped	5259	15673	2031	12638		
		ratio (%)	99.305	97.929	99.732	98.330	0.426	0.401
F022	Ancestral Beijing	mapped	1162270	1143243	1166545	1147960		
		unmapped	26600	45627	22325	40910		
		ratio (%)	97.763	96.162	98.122	96.559	0.360	0.397
J090	Ancestral Beijing	mapped	490815	484340	492424	486326		
		unmapped	5153	11628	3544	9642		
		ratio (%)	98.961	97.655	99.285	98.056	0.324	0.400
J002	Ancestral Beijing	mapped	736473	727044	739288	730539		
		unmapped	5757	15186	2942	11691		
		ratio (%)	99.224	97.954	99.604	98.425	0.379	0.471
J029	Modern Beijing	mapped	953792	936476	957539	941221		
		unmapped	10220	27536	6473	22791		
		ratio (%)	98.940	97.144	99.329	97.636	0.389	0.492
F076	Modern Beijing	mapped	532526	519473	534742	522431		
		unmapped	16374	29427	14158	26469		
		ratio (%)	97.017	94.639	97.421	95.178	0.404	0.539
J111	Modern Beijing	mapped	719693	708076	722895	712304		
		unmapped	14341	25958	11139	21730		
		ratio (%)	98.046	96.464	98.482	97.040	0.436	0.576
	ii) Comparison of mappping frequency ratio (%) among the MTBC lineanges							
		EAI	Haarlem, LAM, X etc.	other non-Beijing				
	Haarlem, LAM, X etc.	ns	-	-				
	other non-Beijing	ns	ns	-				
	Beijing	P < 0.05	ns	ns				

In this analysis CS based on 13 *M. tuberculosis* strains (Table 1) was used as the consensus sequence. i) After mapping with Bowtie2 [42] against H37Rv or CS, the idxstats command of SAMtools [43] was used to calculate the mapping efficacy (Table 5). In read mapping with Bowtie2, both of local and end-to-end mapping mode were performed, and the other parameters were set with default values. Significant differences in mapping frequencies were assessed using multiple comparisons of proportions tests [44]. For all isolates, the difference between H37Rv and CS as a reference differed significantly (p < 0.0001). For both mapping modes, the ratio of mapped to total reads was calculated, and these values used to calculate differences in mapping frequency between the consensus and H37Rv sequences by simple subtraction.

ii) Based on the difference in mapping frequency in 1), the mapping frequencies of MTBC lineages were compared using Mann–Whitney U tests. Combination of Beijing and EAI sequences showed the significan difference (p < 0.05) in mapping frequencies when compared relative to the consensus and H37Rv sequences, the latter belonging to the Haarlem, LAM, X etc. lineage (linage 4).

### Comparison of Illumina read mapping efficacy

Mapping of sequence reads from WGS, such as Illumina, to a reference genome sequence is the first and most crucial step in detecting SNPs and indels in isolates of interest, and for subsequent phylogenetic analysis with SNP concatenated sequences. To compare the mapping results using the consensus and H37Rv sequences as the reference, approximately one million 251 bp x 2 pair-end reads were obtained from clinical *M. tuberculosis* isolates of different MTBC lineages (Tables 5 and 6). About a million reads per isolate were used for the analyses. First, we used Bowite2 [42] and SAMtools [43], which is well established read mapping tool and analysis tool for the resulting mapping, respectively. After mapping with Bowtie against H37Rv or CS, the idxstats command of SAMtools was used to calculate the mapping efficacy (Table 5). Significance tests for multiple comparisons of proportions [44] indicated that each combination of consensus and H37Rv sequences for individual read data in each mapping stringency setting showed significant difference in the proportion of mapped reads (p < 0.0001), with the proportion always higher using CS as the reference. To compare the ratio of reads mapped to unmapped, ratio of mapped reads to total reads (%) in mapping with H37Rv as the reference was subtracted from that of CS. In both of local and end-to-end mapping mode of bowtie, the mapping ratio with CS is better than that of H37Rv.

**Table 6:** **Comparison of Illumina read mapping efficacy using clinical isolates derived from different lineages using**

**Isolate**	**Lineage**		**H37Rv**			**Consensus sequence**			**Subtraction of ratio (%)**		
		Mapping stringency*	**Ambiguity**	**Medium**	**Strict**	**Ambiguity**	**Medium**	**Strict**	**(Consensus-H37Rv)**		
F092	EAI	mapped	676219	676007	674779	677079	676941	676114			
		unmapped	20275	20487	21715	19415	19553	20380			
		ratio (%)	97.089	97.059	96.882	97.212	97.193	97.074	0.123	0.134	0.192
J156	EAI	mapped	1656675	1656191	1652024	1661496	1660887	1657869			
		unmapped	37151	37635	41802	32330	32939	35957			
		ratio (%)	97.807	97.778	97.532	98.091	98.055	97.877	0.285	0.277	0.345
F038	Haarlem, LAM, X etc.	mapped	985717	985256	978713	986844	986318	980541			
		unmapped	43761	44222	50765	42634	43160	48937			
		ratio (%)	95.749	95.704	95.069	95.859	95.808	95.246	0.109	0.103	0.178
F070	Haarlem, LAM, X etc.	mapped	847048	846879	844774	847486	847257	845485			
		unmapped	21212	21381	23486	20774	21003	22775			
		ratio (%)	97.557	97.537	97.295	97.607	97.581	97.377	0.05	0.044	0.082
J073	Haarlem, LAM, X etc.	mapped	1511361	1511205	1508937	1512926	1512725	1511328			
		unmapped	25005	25211	27479	23490	23691	25088			
		ratio (%)	98.372	98.359	98.211	98.471	98.458	98.367	0.099	0.099	0.156
J147	Haarlem, LAM, X etc.	mapped	835484	835319	834077	835192	834976	834227			
		unmapped	14694	14859	16101	14986	15202	15951			
		ratio (%)	98.272	98.252	98.106	98.237	98.212	98.124	−0.034	−0.04	0.018
F081	other non-Beijing	mapped	984775	984303	981542	986321	986079	983479			
		unmapped	31067	31539	34300	29521	29763	32363			
		ratio (%)	96.942	96.895	96.623	97.094	97.07	96.814	0.152	0.175	0.191
J020	other non-Beijing	mapped	1061748	1061460	1059770	1063613	1063346	1062167			
		unmapped	22918	23206	24896	21053	21320	22499			
		ratio (%)	97.887	97.861	97.705	98.059	98.034	97.926	0.172	0.174	0.221
J027	other	mapped	741045	740904	739801	742516	742329	741694			
	non-Beijing	unmapped	13721	13862	14965	12250	12437	13072			
		ratio (%)	98.182	98.163	98.017	98.377	98.352	98.268	0.195	0.189	0.251
F022	Ancestral Beijing	mapped	1140373	1140107	1137877	1145147	1144862	1143607			
		unmapped	32673	32939	35169	27899	28184	29439			
		ratio (%)	97.215	97.192	97.002	97.622	97.597	97.49	0.407	0.405	0.488
J090	Ancestral Beijing	mapped	2087545	2086983	2082777	2095411	2094879	2092249			
		unmapped	47551	48113	52319	39685	40217	42847			
		ratio (%)	97.773	97.747	97.55	98.141	98.116	97.993	0.368	0.37	0.444
J002	Ancestral Beijing	mapped	725501	725308	724182	727822	727702	727147			
		unmapped	13427	13620	14746	11106	11226	11781			
		ratio (%)	98.183	98.157	98.004	98.497	98.481	98.406	0.314	0.324	0.401
J029	Modern Beijing	mapped	935765	935598	934129	939368	939185	938425			
		unmapped	21607	21774	23243	18004	18187	18947			
		ratio (%)	97.743	97.726	97.572	98.119	98.1	98.021	0.376	0.375	0.449
F076	Modern Beijing	mapped	523546	523438	522478	526300	526150	525618			
		unmapped	17480	17588	18548	14726	14876	15408			
		ratio (%)	96.769	96.749	96.572	97.278	97.25	97.152	0.509	0.501	0.58
J111	Modern Beijing	mapped	703968	703761	702412	708028	707765	707024			
		unmapped	17244	17451	18800	13184	13447	14188			
		ratio (%)	97.609	97.58	97.393	98.172	98.135	98.033	0.563	0.555	0.639
ii) Comparison of mappping frequency ratio (%) among the MTBC lineanges											
		EAI	HaarlemLAM, X etc.	other non-Beijing							
	Haarlem,LAM, X etc.	ns	-	-							
	non-Beijing	ns	ns	-							
	Beijing	P<0.05	P<0.01	P<0.05							

In this analysis CS based on 13 *M. tuberculosis* strains (Table 1) was used as the consensus sequence. i) The effects on mapping efficacy were tested for three combinations of parameters: mismatch cost, insertion cost, deletion cost, matching length and similarity. *Mapping stringency was defined as Ambiguous, with frequencies of mismatch cost, insertion cost, deletion cost, matching length and similarity of 2, 2, 2, 0.5, and 0.8, respectively; Medium, with frequencies of 2, 3, 3, 0.5, and 0.8, respectively; and Strict, with frequencies of 3, 3, 3, 0.5, and 0.95, respectively. Significant differences in mapping frequencies were assessed using multiple comparisons of proportions tests [44]. For all isolates, the difference between H37Rv and CS as a reference differed significantly (p < 0.0001). For each stringency setting, the ratio of mapped to total reads was calculated, and these values used to calculate differences in mapping frequency between the consensus and H37Rv sequences by simple subtraction.

ii) Based on the difference in mapping frequency in 1), the mapping frequencies of MTBC lineages were compared using Mann–Whitney U tests. The Haarlem, LAM, X and Beijing sequences showed the greatest difference (p < 0.01) in mapping frequencies when compared relative to the consensus and H37Rv sequences.

Based on a number of typing methods, MTBC could be divided into several lineages [7,12,45]. To analyze whether these MTBC lineages influence the read mapping efficacy, we analyzed the values obtained by subtracting the ratio of mapped to total reads number on the H37Rv sequence from that on CS (Table 5 ii). Using the Mann–Whitney *U* test, we found that isolates from the Beijing family showed significantly greater differences in mapping frequencies between H37Rv and consensus sequences than did isolates from other lineages (P < 0.05), indicating the greater suitability of CS in assessing mapping frequency.

Then we used a commercial software, CLC genomics workbench (CLC bio) to evaluate read mapping efficacy with a different algorism, which is based on Smith and Waterman [46], from Bowtie2. After quality based trimming described in the method section, the sequence reads were mapped to the consensus and H37Rv sequences, and the number of reads mapped and unmapped were compared as did with Bowtie2 and SAMtools. The efficacy of mapping was compared by analyzing three combinations of parameters: mismatch cost, insertion cost, deletion cost, matching length and similarity. Significance tests described above showed that each combination of consensus and H37Rv sequences for individual read data in each mapping stringency setting differed significantly difference in the proportion of mapped reads (p < 0.0001), with the proportion always higher using CS as the reference. The one exception was the isolate J147, which belongs to the Haarlem lineage, as does H37Rv, explaining the higher mapping efficacy found with H37Rv as the reference.

Again, we analyzed whether these MTBC lineages influence the read mapping efficacy in the different methods (Table 6 ii). We found that isolates from the Beijing family showed significantly greater differences in mapping frequencies between H37Rv and consensus sequences than did isolates from other lineages (P < 0.05). The difference was greatest when comparing the Beijing and Haarlem lineages, with the latter being the lineage to which H37Rv belongs (P < 0.01).

The reads failed to map H37Rv specifically and CS specifically were analyzed for their character with contigs constructed by de novo assembling them and BLAST search on the database. Notably, no contigs were created from reads failed to map specifically, indicating the reads were relatively low quality reads to be assembled. BLAST analyses of resulting contigs from reads failed to map H37Rv showed that all contigs had very little identity within the genome sequence of H37Rv as expected, and some of them were MTBC lineage specific while the others were simply missing from genome sequence of H37Rv but not lineage specific.

These results indicate that read mapping of MTBC based on the WGS data is sensitive to both the reference sequence and the MTBC lineage. Our CS provided a better standard for mapping efficacy of different MTBC lineages than did the H37Rv sequence, as well as statistically significant improvements in SNP detection and read mapping, suggesting that CS is a virtually better approach for MTBC research.

### Virtual VNTR typing, spoligotyping and LPS typing

**Figure 3:**
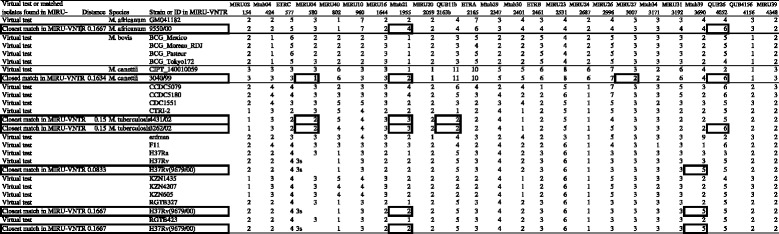
**Virtual VNTR profile of each genome and the similarity search in MIRU-VNTR.** The number of each VNTR marker in each genome was analyzed based on primer sequences [45]. These numbers were input for VNTR analyses by MIRU-VNTR (http://www.miru-vntrplus.org/). The closest match is presented immediately below the line for the corresponding isolate. Markers differing in number for the analyzed strain and its closest match are indicated in bold boxes.

We performed VNTR typing of the 19 mycobacterial strains *in silico* using the MIRU-VNTR 24-loci system [14], which is used commonly in epidemiologic studies of mycobacteria. Based on the reported primer sequence [45], the number of corresponding regions was analyzed in each strain (Figure 3). Of these 19 strains, only the profile for H37Rv was available in the MIRU-VNTRplus database. Comparison of our virtual and actual VNTR profiles of H37Rv showed that 23 of the 24 loci were identical, with one mismatch observed in VNTR3690. This discrepancy may have been due to maintenance of H37Rv stocks in different laboratories [47,48]. When we compared the virtual and actual [49] VNTR profiles of *M. tuberculosis* strain CTRI-2, we found that 20 of the 24 loci (83%) were identical, whereas the other four loci differed slightly, by one copy per locus (Figure 3). Similarly, the virtual and actual profiles of *M. africanum* and *M. canettii* differed in 2 of 24 and 4 of 24 loci, respectively. At present we do not know whether the 2 strains of each species, one tested and the other found in the database, are identical. Thus, these discrepancies may have been due to genetic differences between isolates. Nevertheless, these results suggest that virtual VNTR analysis based on genome sequences is in good agreement with experimental data.

**Figure 4:**
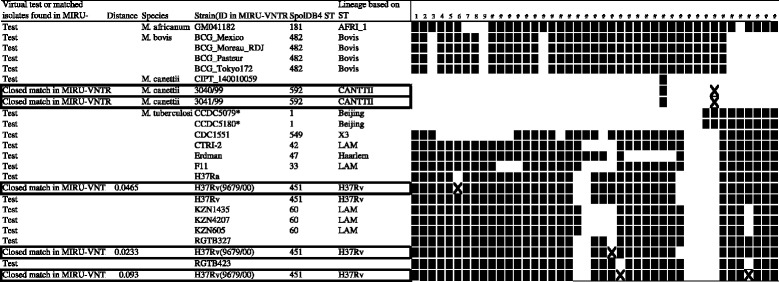
**Virtual spoligotyping of each strain and similarity search at MIRU-VNTR.** Each strain's genome was analyzed for the presence of absence of each spoligo spacer based on primer sequences [50]. The presence or absence of each spacer input for spoligotyping analysis at MIRU-VNTR (http://www.miru-vntrplus.org/). The closest match by similarity search is shown immediately below the line for the corresponding isolate. Markers differing in presence or absence in the analyzed strain and its closest match are indicated in bold boxes. * reported as Beijing type [56].

We also performed spoligotyping *in silico*, a method based on 43 direct repeat (DR) spacer sequences [50] and commonly used in epidemiological studies of mycobacteria. The virtual spoligotype profile of H37Rv was identical to the actual profile stored in the MIRU-VNTRplus database (Figure 4). Of the 19 strains of mycobacteria tested, 15 were correctly grouped into reasonable lineages, whereas *M. canettii*, and *M. tuberculosis* strains H37Ra, RGTB327 and RGTB423 did not yield exact matches. Closer analysis showed that the profile of *M. tuberculosis* strain RGTB423 differed from that in the MIRU-VNTRplus database by two DR markers, whereas the three other strains differed by one DR marker each.

Long sequence polymorphisms (LSPs) were introduced to determine whether lineages of *M. tuberculosis* were associated with specific human populations [7,8]. Utilizing LSPs, MTBC could be divided into at least 6 phylogeographical lineages, each associated with specific, sympatric human populations. LSP analysis was performed on the 19 sequenced genomes of mycobacteria *in silico*, using primers based on target sequence [8] (Table 7). Fifteen strains were classified as lineage 4 (Euro-American lineage) while CCDC5079 and CCDC5180 were classified lineage 2 (East-Asia lineage) and *M. africanum* was classified as lineage 6 (West-Africa lineage).

**Table 7:** **Virtual analyses of LSP and Beijing typing**

**Species**	**Strain name**	**LSP lineage**	**Beijing typing**	
			**Beijing**	**Modern/Ancestral**
*M. tuberculosis*	CCDC5079	2	No	-
	CCDC5180	2	Yes	Modern
	CDC1551	4	No	-
	CTRI-2	4	No	-
	Erdman	4	No	-
	F11	4	No	-
	H37Ra	4	No	-
	H37Rv	4	No	-
	KZN605	4	No	-
	KZN1435	4	No	-
	KZN4207	4	No	-
	RGTB327	4	No	-
	RGTB423	4	No	-
*M. bovis* BCG	Mexico	4	No	-
	Moreau RDJ	4	No	-
	Pasteur	4	No	-
	Tokyo 172	4	No	-
*M. africanum*	GMO41182	6	No	-
*M. canettii*	CIPT 140010059	2	No	-

LSP analysis [8] and Beijing typing [55] of target sequences were performed *in silico* using the indicated primers in the articles.

Cross-sectional studies in diverse geographic locations have shown epidemiologic associations between Beijing types of *M. tuberculosis* and increased risks of drug resistance [51]. Beijing typing was originally based on spoligotyping [52], but was later determined by detection of specific SNP [53,54] and PCR analysis of the insertion of IS6110 into specific positions [55]. We analyzed whether the 19 sequenced MTBC genomes could be classified as Beijing type, based on IS6110 insertion between Rv0001 and Rv0002, as shown for the H37Rv genome; or into a modern or ancestral subtype based on IS6110 insertion into the NTF region [55] (Table 7). Of the strains tested, only CCDC5180 was Beijing type. Although spoligotyping indicated that CCDC5079 should also be Beijing type (Figure 4 and [56]), this strain lacks an inserted IS6110 between Rv0001 and Rv0002. Although classification of Beijing type based on IS6110 insertion is limited in classifying MTBC lineages, virtual Beijing typing could be performed using an *in silico* approach.

Taken together, these results suggest that virtual VNTR, spoligotyping, LSP analysis and Beijing typing *in silico* can be utilized for epidemiological analysis of mycobacterial strains without the need for PCR amplification and/or hybridization procedures.

## Discussion

In this study, we successfully aligned whole genome sequences of 19 MTBC strains by correcting the large rearrangements found in the genomes of KZN605, KZN1435 and KZN4207, and using very fast multiple sequence alignment software, MAFFT [32,57]. This alignment allowed us to create a virtual consensus genome sequence of MTBC, reflecting all genetic information from various lineages. In comparing this CS with that of H37Rv as the reference sequence, we found that CS allowed an unbiased and efficient detection of critical SNPs, distinguishing among the lineages of MTBC. Use of CS as a reference reduced the SNP calling bias, as shown for *M. canettii*. Moreover, SNP concatemers of MTBC strains based on CS were better able to reproduce a phylogenetic tree based on whole genome alignment than concatemers based on H37Rv. Phylogeny of *M. tuberculosis* is very closely related the human evolution, and consistent with MTBC displaying characteristics indicative of adaptation to both low and high host densities [20]. Ford at al reported that *M. tuberculosis* strains from lineage 2 (East Asian lineage and Beijing sublineage) acquire drug resistances *in vitro* more rapidly than *M. tuberculosis* strains from lineage 4 (Euro-American lineage) and that this higher rate can be attributed to a higher mutation rate [51]. Thus precise and accurate phylogenetic analysis based on SNP concatemers is becoming a key importance of *M. tuberculosis* research. Using H37Rv as a reference to call SNP led to some inadequate clustering of *M. tuberculosis* strain. As shown in Figure 1c, RGTB432 becomes an out-group although it should be placed on the lineage 4 group as in Figure 1a. Thus, adequate reference sequence is not only important for efficient analysis such as mapping of read data from next generation sequencers but also phylogenetic analysis based on SNP concatemers because it directly link to evolution and drug resistance of *M. tuberculosis* strain. Use of CS also significantly reduced the total number of SNPs detected, decreasing computational time by an order of magnitude. Reduction of computational time is extremely useful when analyzing a large number (more than hundreds) of isolates.

During the construction of CS, we also called SNPs and indels using as reference the H37Rv genome to update information about polymorphisms found in MTBCs. Complete SNP data of individual isolates were presented with position and annotations. As reported previously, about 50% of the indels were located in the genes encoding the PE-PGRS and PPE family of proteins while about 25% were located in intergenic regions. The positions, length, annotation and strains of all SNPs and indels have been reported for further applications, such as exploration of lineage specific gene traits.

Use of CS as a reference also significantly improved the efficacy of short-read mapping of clinical isolates. Use of a particular strain as the reference can affect the mapping results, depending on the lineage of that strain. Testing of isolates of a different lineage from the reference strain can result in the omission of some SNPs and/or indels critical for further analysis. Use of a consensus sequence as a reference would minimize this possibility.

VNTR typing, spoligotyping, LSP analysis and Beijing typing. Thus, technically, it is possible to perform these analyses using sequence data of MTBC strains. To prove this concept, we performed these analyses *in silico*. Although actual typing data were available for a few of the strains tested, we observed fairly good agreement between actual and expected data. Virtual typing also showed several limitations. For example, Beijing typing classified strain CCDC5079, which belongs to the Beijing family, as non-Beijing type based on IS6110 insertion. Thus, one typing method would not be sufficient to accurately type MTBC isolates. As WGS technologies improve, SNP concatenation would become the ideal typing method. Moreover, we found that *in silico* analysis using CS was highly reproducible and robust because of its intrinsically objective nature, an objectivity sometimes lacking during actual epidemiological analysis of MTBC. *in silico* analysis is also labor-saving, since it requires only WGS data.

Our consensus genome sequence does not contain sequence information on several lineages, including lineages 1, 3 and 5 in LPS analysis. This could be a potential shortage because lacks of particular lineages in CS could lead bias in calling SNP as shown in this study. At present, few complete whole genome sequences of these lineages are available in the database. We intend to update our consensus sequence when such information becomes available.

## Conclusion

We generated virtual consensus sequences of MTBC from 13 *M. tuberculosis* and 6 non-tuberculosis strains, and showed that this sequence was superior to the H37Rv sequence as a reference in MTBC research. A completely annotated consensus sequence, relative to the sequence of H37Rv, is available as the additional data. Construction of a web based service integrating the phylogenetic and epidemiological analyses performed in this study is currently underway.

## Methods

### Multiple genome sequence alignment and construction of a virtual MTBC consensus genome sequence

Whole genome sequences used to construct CSs in this study are detailed in Table 1. Genome sequences of 19 strains available by April 2012 were used: 13 genomes from *M. tuberculosis*, one each from *M. africanum* and *M. canetti,* and four from *M. bovis* BCG. Genome rearrangement was analyzed by a publicly available software, Mauve [31], with large inversions, hampering genome alignment, observed in *M. tuberculosis* strains KZN605, KZN1435 and KZN4207 and manually corrected. We constructed two types of consensus whole genome sequences: one containing sequence data from 13 *M. tuberculosis* strains and the other one containing sequence data from all four species of mycobacteria (19 strains). All sequence data were downloaded from the NCBI databases and aligned using publicly available alignment software, MAFFT version 6 or 7 [32,57] (http://mafft.cbrc.jp/alignment/server/) using our own-build Linux-based server. Consensus sequences were constructed by merging the alignment results using a sequence editing commercial software, Genetyx (Genetyx Inc, Tokyo Japan). Two types of consensus sequences were prepared with handling SNPs as majority rule or ambiguity rule. In this study, ambiguity sequence was used for further analysis.

### Annotation of the virtual *M. tuberculosis* consensus genome

The consensus genome, consisting of an artificially assembled sequence, was annotated manually and edited using a commercial genome sequence editing software, *in silico* molecular cloning (in silico Biology Co., Kanagawa, Japan). Because CS used for the annotation contains many ambiguous nucleotides and insertion, instead of CDS extraction, homologous regions based on corresponding nucleotide sequences of CDS in H37Rv was determined to extract the corresponding regions in CS, with the assignment of each region based on the locus_tag (the Rv number) of CDS, repeat regions, rRNA and tRNA of H37Rv.

### SNP and indel extraction, annotation and characterization

Each genome sequence of MTBC listed in Table 1 was compared with the constructed CS and with the genome sequence of *M. tuberculosis* H37Rv using a publicly available software, MUMmer 3.0 [58]. SNPs and indels were extracted from each MTBC strain. For SNP analysis, insertions of more than two bp and all deletions were excluded from the resulting data. The resulting files from MUMmer were converted into the VCF format to annotate the SNPs using the a commercial software, CLC genomics workbench (CLC bio). Since the algorithms used to align genome sequences for the construction of the consensus genome (MAFFT) and for the extraction of SNPs and indels (MUMmer) are different, we manually checked the result of indel calls in the MUMmer data, and indels greater than 5 bp were used for manual annotation.

### Phylogenetic analysis using SNP concatenated and whole genome sequences

Phylogenetic trees were constructed from SNP and whole-genome sequence alignments. Two methods were used to evaluate robustness: a maximum-likelihood approach, PhyML 3.0 [39] and the Bayesian MCMC framework, BEAST1.7 [38]. For PhyML, GTR and gamma were chosen for a nucleotide substitution model, and tree robustness was evaluated by two methods: approximate likelihood-ratio test (aLTR) [37] implemented in PhyML. Bootstrappings implemented in PhyML was used to generate multiple trees (100 trees for SNP concatemers) for choosing most probable trees based on combination of 9 statistical analyses using CONSEL [40]. Because data size limitation in CONSEL, 40 trees were generated by PhyML for phylogenetic analysis with whole genome alignment.

For BEAST, various combinations of population size change and molecular clock models were compared to find the model that best fit the data. A simple HKY model was used for SNP concatemer based phylogenetic trees, whereas an HKY kappa model was used for whole-genome sequence based phylogenetic trees. MCMC chains were run for 10 million generations, with sampling every 1000th generation. Convergences and Effective Sample Sizes (ESSs) of the estimates were checked using Tracer v1.4 (http://beast.bio.ed.ac.uk/Tracer). Three of the phylogenetic trees we constructed had ESS values greater than 100, suggesting sufficient mixing of the Markov chain. Maximum clade credibility (MCC) trees were created and annotated using TreeAnnotator within the BEAST software package. All analyses were performed on Linux or Windows 7 based computers. Trees were visualized with FigTree (http://tree.bio.ed.ac.uk/software/figtree/). The tree to tree distance were compared using Robinson and Fould test [41] implemented in the treedist program in the Phylip package (http://evolution.genetics.washington.edu/phylip/doc/treedist.html) with both of unrooted and rooted mode. The computation time for each analysis was obtained from the log files. Phylogenetic data related to this study have been registered at TreeBase (http://datadryad.org/(doi:10.5061/dryad.nq070)).

### Comparison of Illumina read mapping efficacy

To compare the mapping efficacy using CS as reference with H37Rv, we obtained approximately one million 251 bp x 2 pair-end reads from clinical *M. tuberculosis* isolates listed in Tables 5 and 6 using MiSeq with Nextera XT library kits (Illumina). The sequence data used have been registered with DNA Data Bank of JAPAN (DDBJ) as accession number DRA001219. First, Read mapping was performed using Bowtie 2 [42] with local mode and end-to-end modes and default parameters. The resultant mapping was analyzed the idxstats coomand of SAMtools [43]. We also analyzed the mapping efficacy using CLC genomics workbench, of which algorithm is based Smith and Waterman [46], after trimming based on base quality (quality score limit = 0.05, removing reads if there are more than 2 ambiguous nucleotides in the reads or less than 15 bp in length). In the analysis with CLC genomics workbench, the influence of three combinations of parameters on mapping was tested: mismatch cost, insertion cost, deletion cost, matching length and similarity. In both analyses, significance of differences in mapping frequencies were assessed using multiple comparisons of proportions tests [44]. We also compared ratio of the number of reads mapped to total read for the two reference sequences, and subtracted values of the mapping frequencies (% mapped reads with consensus sequence minus that of H37Rv) were calculated. The subtracted values were used to compare mapping frequency among the MTBC lineages with Mann–Whitney U tests.

### Virtual VNTR typing, spoligotyping, LSPs typing and Beijing typing

Based on the amplification primers for 24-loci VNTR [6], spoligotyping [50], LSPs [7,8], and Beijing typing based on the insertion positions of IS6110 [55], sequence data corresponding to the respective loci or regions were selected. Using the Blast algorithm, these sequence data were used to analyze whether each region is present in the MTBC strains listed in Table 1 [59]. VNTR and spoligotyping results for the isolates or strains in the database were analyzed and compared on the MIRU-VNTR web site (http://www.miru-vntrplus.org/MIRU/index.faces) [45].

## Additional files

Additional file 1:
**Consensus sequence of MTBC with SNP handled by majority rule.**
Additional file 2:
**Consensus sequence of MTBC with SNP handled by ambiguity rule.**
Additional file 3:
**CS with SNP handled by ambiguity rule and annotation based on H37Rv.**
Additional file 4:
**SNP distribution among MTBC in comparison with H37Rv.** Description of data: SNPs found in each MTBC were summarized with their distribution among the strains and location of the SNPs in the genes. Additional file 5:
**Indels distribution among MTBC in comparison with H37Rv.** Description of data: Indels more than 5 bp in the length found in each MTBC were summarized with their distribution among the strains and location of the SNPs in the genes. Additional file 6:
**Maximum-likelihood phylogenies based on whole genome and SNP concatenated sequence alignment.** Description of data: Phylogenetic trees based on whole genome sequence (a), SNP concatemers using the consensus genome sequence as reference (b) and SNP concatemers using H37Rv genome as reference (c) were constructed using PhyML [37]. Multiple trees (100 trees for a and b, 40 trees for c) were generated by boot-strapping in PhyML analysis, and most probable trees were selected by a combination of 9 statistical methods implemented in CONSEL [40]. Isolates, clustered into different positions compared with the phylogenetic tree based on the whole genome sequences of *M. tuberculosis*. For the KZN series, inversion-corrected sequences were used for the alignment and marked “_m”. Additional file 7:
**Probability value (i.e., p-value) to assess the confidence in the selection problem of phylogenetic tree using CONSEL.** Description of data: Using PhyML with 100 (for SNP concatemers) or 40 (whole genome (CS) bootstrapping, multiple trees generated were analyzed with CONSEL to select most probable tree. P-values of top 10 trees generated from PhyML are indicated and the most probable trees in the analyses were used to generate Additional file 6: Figure S1. Abbreviation of the statics analyzed in CONSEL; obs: the observed log-likelihood difference. au: the p-value of the approximately unbiased test calculated from the multiscale bootstrap. np: the bootstrap probability calculated from the multiscale bootstrap. bp: the bootstrap probability calculated in the usual manner. kh: the Kishino-Hasegawa test. sh: the Shimodaira-Hasegawa test. wkh: the weighted Kishino-Hasegawa test. wsh: the weighted Shimodaira-Hasegawa test.
