# Supplementary figures and images for: Construction of a virtual Mycobacterium tuberculosis consensus genome and its application to data from a next generation sequencer
Source: BMC Genomics. 2015 Mar 20;16(1):218. doi: 10.1186/s12864-015-1368-9 (PMC4425900; doi:10.1186/s12864-015-1368-9)

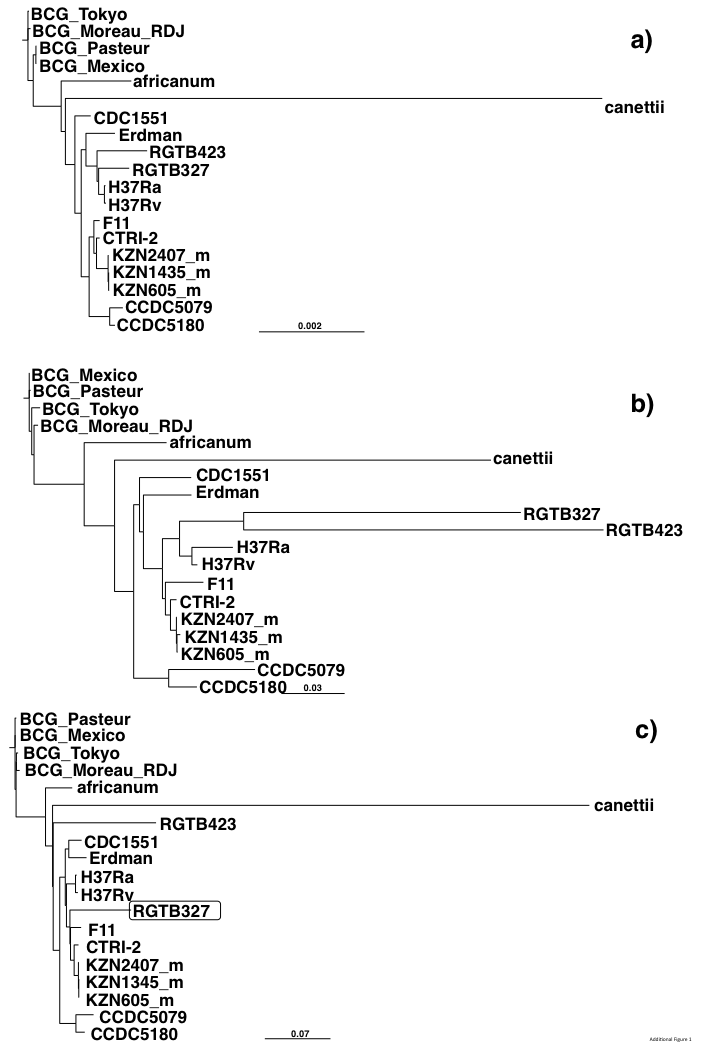

Supplement: Additional file 6: — Maximum-likelihood phylogenies based on whole genome and SNP concatenated sequence alignment. Description of data: Phylogenetic trees based on whole genome sequence (a), SNP concatemers using the consensus genome sequence as reference (b) and SNP concatemers using H37Rv genome as reference (c) were constructed using PhyML [37]. Multiple trees (100 trees for a and b, 40 trees for c) were generated by boot-strapping in PhyML analysis, and most probable trees were selected by a combination of 9 statistical methods implemented in CONSEL [40]. Isolates, clustered into different positions compared with the phylogenetic tree based on the whole genome sequences of M. tuberculosis. For the KZN series, inversion-corrected sequences were used for the alignment and marked “_m”. [file 12864_2015_1368_MOESM6_ESM.tiff]
